# Approach to determine the diversity of *Legionella* species by nested PCR-DGGE in aquatic environments

**DOI:** 10.1371/journal.pone.0170992

**Published:** 2017-02-06

**Authors:** Wen-Chien Huang, Hsin-Chi Tsai, Chi-Wei Tao, Jung-Sheng Chen, Yi-Jia Shih, Po-Min Kao, Tung-Yi Huang, Bing-Mu Hsu

**Affiliations:** 1 Department of Medicine, Mackay Medicine College, Taipei, Taiwan, ROC; 2 Department of Thoracic Surgery, Mackay Memorial Hospital, Taipei, Taiwan, ROC; 3 Mackay Junior College of Medicine, Nursing, and Management, Taipei, Taiwan, ROC; 4 School of Medicine Tzu-Chi University, Hualien, Taiwan, ROC; 5 Department of Psychiatry, Tzu-Chi General Hospital, Hualien, Taiwan, ROC; 6 Section of Respiratory Therapy, Cheng Hsin General Hospital, Taipei, Taiwan, ROC; 7 Graduate Institute of Life Sciences, National Defense Medical Center, Taipei, Taiwan, ROC; 8 Department of Earth and Environmental Sciences, National Chung Cheng University, Chiayi, Taiwan, ROC; Leibniz-Institute DSMZ, GERMANY

## Abstract

In this study, we describe a nested PCR-DGGE strategy to detect *Legionella* communities from river water samples. The nearly full-length 16S rRNA gene was amplified using bacterial primer in the first step. After, the amplicons were employed as DNA templates in the second PCR using *Legionella* specific primer. The third round of gene amplification was conducted to gain PCR fragments apposite for DGGE analysis. Then the total numbers of amplified genes were observed in DGGE bands of products gained with primers specific for the diversity of *Legionella* species. The DGGE patterns are thus potential for a high-throughput preliminary determination of aquatic environmental *Legionella* species before sequencing. Comparative DNA sequence analysis of excised DGGE unique band patterns showed the identity of the *Legionella* community members, including a reference profile with two pathogenic species of *Legionella* strains. In addition, only members of *Legionella pneumophila* and uncultured *Legionella* sp. were detected. Development of three step nested PCR-DGGE tactic is seen as a useful method for studying the diversity of *Legionella* community. The method is rapid and provided sequence information for phylogenetic analysis.

## Introduction

In developing countries, about 80% of diseases and one-thirds of fatal cases are associated with waterborne pathogens [1]. As a potential threat to public health, *Legionella* species are omnipresent in the natural aquatic environments, for example, river, lake, hot spring, and drinking water. Under specific environmental conditions, the density of these microorganisms can increase rapidly, as agreed to causing outbreaks of disease. Various systems (e.g., water supplies, cooling towers, hydrotherapic establishments, spa) provide ideal growth conditions and thus represent a worrying source of exposure for humans.

The first reported case of Legionnaires’ disease in Taiwan was in 1985, indicating the lurking infection risk of *Legionella* in domestic aquatic environments [2]. Another significance of free-living amoeba in public health is their potential role as hosts of several pathogenic bacteria including *Legionella* and *Pseudomonas* [3–7]. Because of the presence of endosymbionts like *Legionella* in *Acanthamoeba* mutually increases the toxicity and pathogenicity of each other [8,9]. Controlling the *Legionella* risk in these systems is necessary to protect the population.

In 2006, a French normative association AFNOR (XPT 90–471) concerning the “detection and quantification of *Legionella* and/or *Legionella pneumaphila* by concentration and gene amplification using polymerase chain reaction (PCR)” has been published. It establishes in particular the requirements of PCR methods performances in bacteria detection. Moreover, the detection of *Legionella* spp. has been adopted for cooling tower systems prevention.

Although most of *Legionella spp*. identification using culture and biochemical methods were well documented, many of the others have not been identified included unculturable *Legionella spp*. Contrary to cultural method, molecular techniques are able to estimate the density (real-time qPCR) and the composition (Denaturing Gel Gradient Electrophoresis, DGGE) of a microbial community. Rapidly DGGE is a technology used to profile and identify dominant members of the microbial community based on its ability to separate double stranded DNA amplicons of similar size. The separation of DNA product is based on sequence composition that can be assessed by GC% and melting temperature (Tm) values. The gel contains a gradient of denaturing agents and nucleotide duplexes with different Tm value could be differentiated and excised for sequencing after electrophoresis. The development of these techniques allowed new perspectives in the control of *Legionella* community in aquatic environmental samples. As example, other genomes than *Legionella pneumophila* are now much more frequently detected.

In this study, a specific DGGE method was used to monitor the difficulty in detecting the very low concentrations of *Legionella* species from river water samples. It consists of a reference profile based on pathogenic *Legionella* strains. Our strategy was a three step approach combined with nested PCR followed by DGGE analysis, which included the first step of bacterial common full-length 16S rRNA gene amplification, the second step of *Legionella* specific gene amplification, and the final step of DGGE pattern analysis that consisted with a GC clamp DNA fragment amplification and a DGGE electrophoresis. Instantaneous DGGE analysis of PCR products gained by direct and indirect method completed it possible to suppose the diversity of *Legionella* in the river water samples. Therefore, the aim of this study was to establish the nested PCR-DGGE approach and to test empirically on the river network areas of Puzi River.

## Materials and methods

### River water sample collection and concentration

The total two river water samples were collected along the Puzi River and its approximate geographical coordinates were (23.481883, 120.290867) and (23.487250, 120.26693). The sample collections were carried out at November 2015 and its temperature were about 21°C. Puzi river is a publicly accessible site, which specific permission for collecting water samples was not required. About 1 L of water was taken right beneath surface transported to the laboratory in 1 L sterile bottles at 4°C within 12 h. For concentration of microorganisms, 1 L sample water was filtered through 45 mm GN-6 metricel membrane disc filter (Pall, Mexico) with 0.45 μm stainless steel filter holder. The membranes were then eluted with 50 ml of phosphate buffered saline. The eluent was transferred into 50 ml centrifuge tubes and centrifuged at2,600×g for 30 min. For centrifuged solution, the top supernatant fluid of 40 ml was removed, and the remaining 10 ml concentrate was centrifuged again. For secondary centrifuged solution, the top supernatant fluid of 4 ml was removed, and the remaining 1 ml concentrate was used for subsequent experiments.

### DNA extraction

The 1ml concentrate was transferred to an eppendorf. The concentrate was used for extracting DNA using the MagPurix 12s automatic DNA extraction system and MagPurix Bacterial DNA Extraction Kit (Zinexts Life Science, Taiwan). The quantity and quality of DNA was estimated using NanoDrop spectrophotometer.

### Amplification of bacterial 16S rRNA gene using the *Legionella* specific primers

Nested PCR of 16S rRNA gene was performed with DNA extracted from river water samples by using the nearly full-length bacterial primers and *Legionella* specific primers (Table 1). The product gained was used as template DNA for a second PCR with *Legionella* specific primer pair. The result was confirmed on 1% agarose gel electrophoresis. Sterilized ddH_2_O was used as the template DNA for a negative control. *Legionella pneumophila* ATCC 33823 and *Legionella dumoffii* ATCC 33279 were also included as a positive control.

**Table 1:** List of PCR primers used in this study.

Primers set	Sequences	Target gene	Amplicon size	Predenaturation	Denaturation	Annealing	Extension	Final Extension	Cycling no.	Reference
LEG225	AAGATTAGCCTGCGTCCGAT	LEG 16S rRNA	654 bp	94°C/15 min	94°C/30s	62°C/60s	72°C/60s	72°C/10 min	35	(Miyamoto et al., 1997)
LEG448	GAGGGTTGATAGGTTAAGAGC	LEG 16S rRNA	410 bp	95°C/1.5 min	65°C/60s	72°C/60s	72°C/60s	72°C/10 min	20	
LEG858	GTCAACTTATCGCGTTTGCT									
GCLEG448	CGCCCGCCGCGCGCGGCGGGCGGGGCGGGGGCACGGGGGGGAGGGTTGATAGGTTAAGAGC	LEG 16S rRNA	410 bp	95°C/1.5 min	95°C/30s	66°C/60s	72°C/30s	72°C/10 min	35	
LEG858	GTCAACTTATCGCGTTTGCT									
E8F	AGAGTTTGATCATGGCTCAG	Nearly full-length 16S rRNA	1500 bp	95°C/7 min	95°C/30s	55°C/30s	72°C/90s	72°C/7 min	30	(Mathew et al., 2015)
U1510R	CGGTTACCTTGTTACGACTT									

### Three step process of nested PCR-DGGE

**Fig 1:**
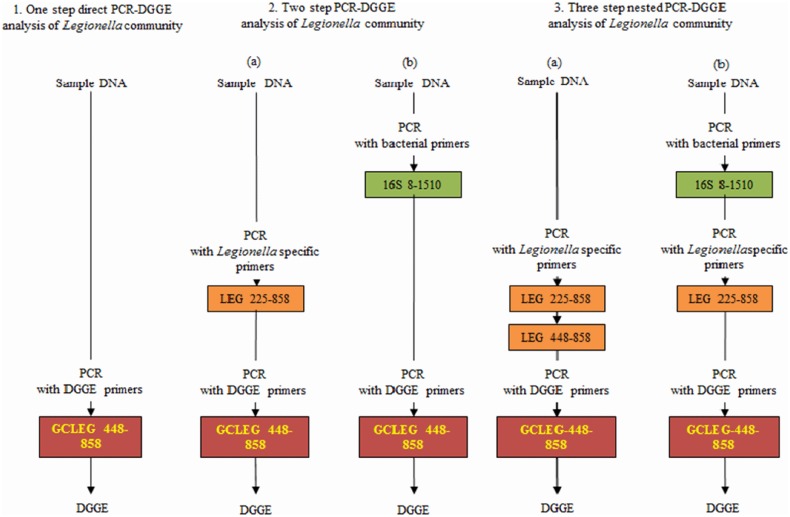
Graphic overview of the various PCR-DGGE tactics used to study the diversity of *Legionella* in river water samples analyzed. (1) single step directed PCR-DGGE tactic; (2) two step PCR-DGGE tactic; (3) three step nested PCR-DGGE tactic. Comparative DGGE pattern analysis of PCR products gained by tactics1/2/3 makes it likely to infer the diversity of *Legionella*.

Five tactics were used to analyze the *Legionella* in the river water samples (Fig 1). First, the 16S rRNA gene fragment was amplified using the GCLEG448 and LEG858 primers (Fig 1.1). Second, the three step nested PCR amplification was completed to gain various *Legionella* specific 16S rRNA gene fragments apposite for DGGE (Fig 1.3). The nearly full-length 16S rRNA fragment was amplified using the E8F and U1510R bacterial primers in the first step. The product gained was used as the template DNA for the second PCR amplification using the *Legionella* specific LEG 225 and LEG858 primers. Eventually, to originate PCR products apposite for DGGE, the third round of PCR amplification was completed with GCLEG448 and LEG858 DGGE primers using the product of a second round as template DNA. The PCR products gained were subjected to DGGE analysis using a Bio-Rad DCode^™^ Universal Mutation Detection System (Bio-Rad, USA). The DGGE gels were composed of 6% polyacrylamide [Acrylamide/Bis-acrylamide 29:1, 40% (w/v) solution; Amresco, USA] and contained 30–40% denaturing gradient (100% denaturing solution contained 40% deionized formamide and 7.0 M Urea). The DGGE was run in 1x TAE buffer (0.5 mM Na_2_EDTA (pH 8.0), 10 mM Na-acetate, 20 mM Tris) at 60°C, 220V, for 3 h. The DGGE gels were stained with TAE buffer/1,000 diluted EtBr for 20 min. Consequently, the gels were photographed using a UV transilluminator. The PCR products for gene sequencing were purified with the GenepHlow^™^ Gel/PCR Kit (Geneaid). The BigDye^®^ Terminator v3.1 Cycle Sequencing Kit and ABI PRISM^®^ 3730xl DNA Analyzer was used for sequence analysis by Mission Biotech, Taiwan. The sequence data has been submitted to GenBank (www.ncbi.nlm.nih.gov) and the assigned accession numbers were from KY311793 to KY311807 and KY451032 to KY451033.

### *Legionella* sequence phylogenetic analysis

The all nucleotide sequences were compared in NCBI GenBank databases using BLAST analysis. Then, the phylogenetic tree analysis was imported into the MEGA5 software by using neighbor-joining method with 1,000 bootstrapped replicates.

## Results

### *Legionella* diversity analysis using nested PCR-DGGE strategies

**Fig 2:**
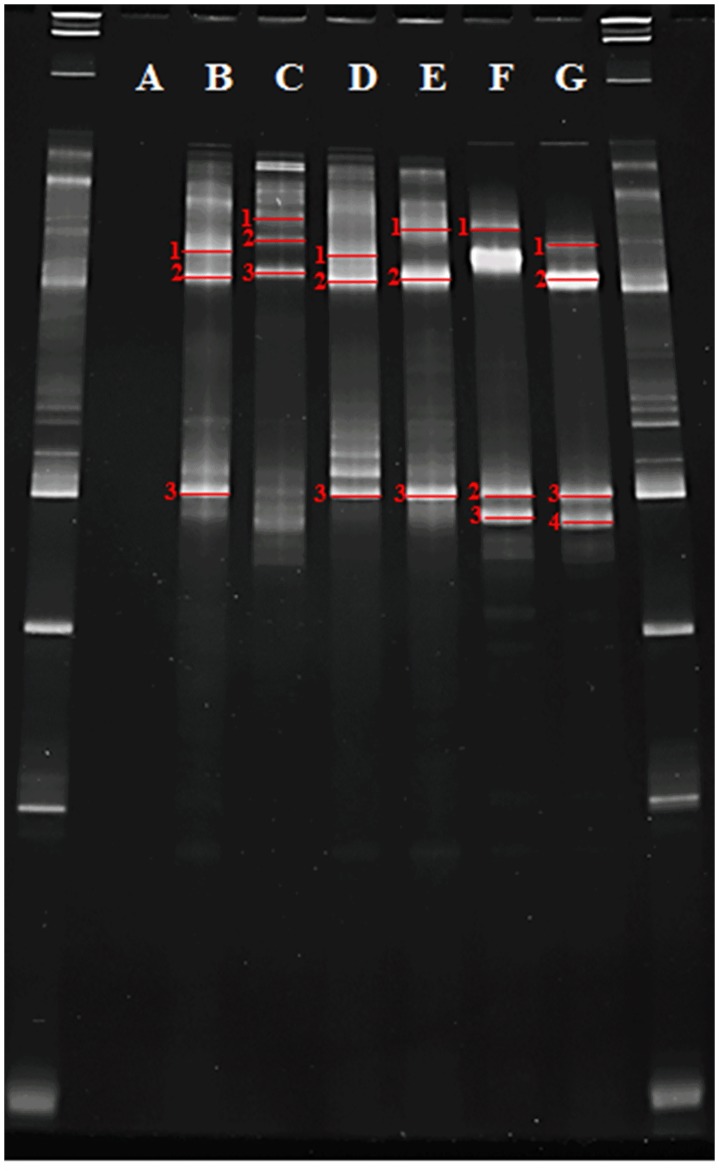
DGGE band patterns of 16S rRNA fragments gained after PCR amplification using various primers and DNA from river water samples. Lanes: A-E, pattern of river water samples; Lanes: F and G, pattern of positive control samples (*Legionella pneumophila* ATCC 33823 and *Legionella dumoffii* ATCC 33279); Lanes: A, F and G, pattern gained with PCR products amplified using single step direct PCR method (strategy 1); Lanes: B and C, pattern gained from the product amplified using the two step PCR method (strategy2); D and E, pattern when primers specific to the *Legionella* species were used in the three step nested PCR method (strategy 3). DGGE patterns that were excised for DNA sequence analysis are numbered.

Five different strategies were applied to analyze the *Legionella* diversity in the river water sample. In the first process (Fig 1.1), DNA was extracted from the river water sample, and the DGGE primer pairs GCLEG448 and LEG858 were used to amplify this DNA to produce LEG 16S rRNA amplicons. These amplicons were used for DGGE analysis.: No bands appeared in lane A of the gel, thus indicating that *Legionella spp*. could not be detected in the river water sample (Fig 2). However, the DGGE also resulted in four other bands that may possibly depict the positive control (*Legionella pneumophila* ATCC 33823 and *Legionella dumoffii* ATCC 33279) in single step PCR-DGGE. In the second process (Fig 1.2), the two-step nested amplification products (*Legionella* specific, bacterial and DGGE primers) from the river water sample were compared to the single-step direct PCR amplification products. The resultant DGGE pattern (Fig 2, lanes B and C) was found not similar to a few bands. In the third process (Fig 1.3), the DGGE pattern produced by analyzing the products of the three-step nested PCR only comprised bands for *Legionella* spp. These fragments were produced using *Legionella* specific primer pairs (LEG225 and LEG858) in the second amplification step to avoid the PCR amplification of non-*Legionella* species. This process also allowed the evaluation of the bacterial-*Legionella*-specific and *Legionella*-specific DGGE pattern of *Legionella* to the *Legionella* community DGGE pattern of the identical river water sample (Fig 2, lane D and E).

### Comparative *Legionella* sequence analyses

**Fig 3:**
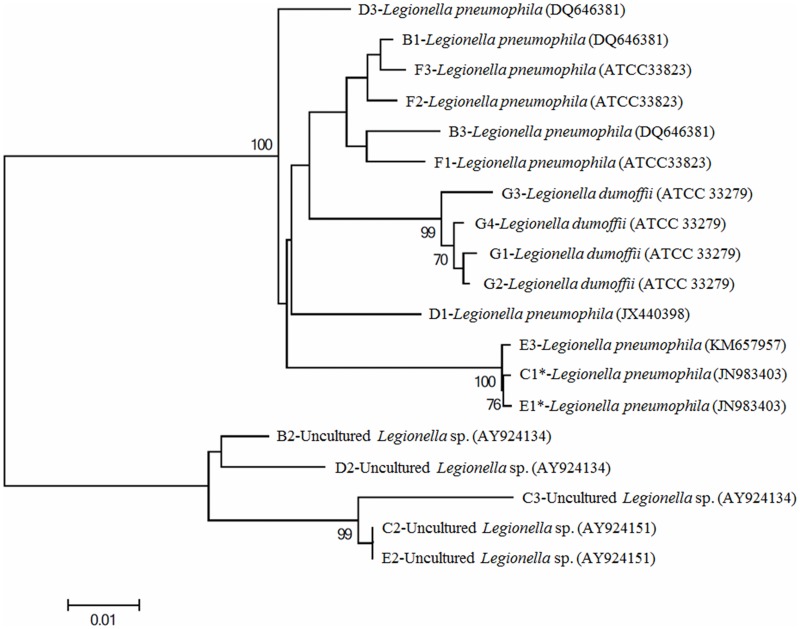
A phylogenetic tree produced with the neighbor-joining method based on the 16S rRNA gene sequences of *Legionella* from river water samples. The numbers of the sequences in this tree refer to the numbers in the DGGE. The scale bar represents 200% of nucleotide sequence divergence. The numbers at the selected nodes indicate the levels of bootstrap support (percentage) based on 1000 re-sampled data sets (only values greater than 70% shown).

The PCR products were sequenced for species identification. Segregated DNA fragments were resected and sequenced to confirm the presence of discriminating *Legionella* species in a water sample from the Puzi River. A total of 19 bands (Fig 2) were resected after DGGE, and their gene sequences were further analyzed. Using the NCBI BLAST sequence analysis tool, the PCR products listed in Table 2 were found to be similar to sequences of *Legionella* species with a high identity value. The identified species include uncultured *Legionella* sp. and *Legionella pneumophila*. Phylogenetic tree analysis supported these results (Fig 3). The gene sequences of DGGE bands B1, B3, and F1 to F3 clustered with the gene sequence of *Legionella pneumophila* DQ646381 (sample) and ATCC 33823 (positive control), respectively. The sequences of DGGE bands B2, C2, C3, D2, and E2 were clustered with the sequence of uncultured *Legionella* sp. (AY924134 and AY924151). The sequences of bands C1, E1, and E3 clustered and were phylogenetically related to *Legionella pneumophila* (JM98403 and KM657957). The sequences of DGGE bands D1 and D3 clustered with the sequence of *Legionella pneumophila* (JX440398 and DQ646381), while the sequences of DGGE bands G1 to G4 clustered with the sequence of positive control *Legionella dumoffii* (ATCC 33279).

**Table 2:** Sequence identity of excised DNA fragments.

Band name with accession no.*	Database match with accession no. in parentheses	% Identity
B1(KY311793)	*Legionella pneumophila* (DQ646381)	99
B2(KY311794)	Uncultured *Legionella* sp. (AY924134)	98
B3(KY451032)	*Legionella pneumophila* (DQ646381)	98
C1(KY311798)*	*Legionella pneumophila* (JN983403)	96
C2(KY311795)	Uncultured *Legionella* sp. (AY924151)	98
C3(KY311796)	Uncultured *Legionella* sp. (AY924134)	97
D1(KY451033)	*Legionella pneumophila* (JX440398)	99
D2(KY311797)	Uncultured *Legionella* sp. (AY924134)	99
D3(KY451032)	*Legionella pneumophila* (DQ646381)	98
E1(KY311798)*	*Legionella pneumophila* (JN983403)	96
E2(KY311799)	Uncultured *Legionella* sp. (AY924151)	98
E3(KY311800)	*Legionella pneumophila* (KM657957)	97
F1(KY311801)	*Legionella pneumophila* (NZ_LAXX00000000)	99
F2(KY311802)	*Legionella pneumophila* (NZ_LAXX00000000)	99
F3(KY311803)	*Legionella pneumophila* (NZ_LAXX00000000)	99
G1(KY311804)	*Legionella dumoffii* (X73405)	99
G2(KY311805)	*Legionella dumoffii* (X73405)	99
G3(KY311806)	*Legionella dumoffii* (X73405)	99
G4(KY311807)	*Legionella dumoffii* (X73405)	99

*This mark means the strain only closest cultivated strain from NCBI (the identification is below 97%)

The Figs and Tables of this study have been placed in website **Figshare**. (https://figshare.com/projects/Approach_to_determine_the_variety_of_Legionella_species_by_nested_PCR-DGGE_in_aquatic_environments/17990)

## Discussion

Nested amplification, a *Legionella*-like product was obtained from the river water sample. Semi-nested, nested and double PCR amplification protocols were used previously to improve the sensitivity of detection [10–15]. Furthermore, the nested PCR method, using degenerate and specific primers were found to increase the detection sensitivity of 100 copies of a gene per reaction [15]. The PCR products gained by the two and three step nested PCR process indicated that members of the *Legionella* were dominant in the river water sample. However, *Legionella* might be present in low concentrations, as these were detected only after by nested PCR method. The improved detection signal strength in the nested PCR may possibly be owing to the first round resulting in the PCR amplification of enough quantities of DNA level from the *Legionella* present in low concentrations and also owing to the dilution of inhibitory materials such as urea, calcium ions, humic acids, and melanin present in the water sample [16–18].

The molecular techniques for detection of environmental microbes by means of DGGE may become hard if they were present in low concentrations, additionally water samples of the river contamination, which comprise of a complex mixture of environmental microbes. The DGGE using bacterial 16S rRNA primer pairs mostly detects the main components of the analyzed bacterial community ignoring the less plentiful but probably important species [19]. Existence of *Legionella* further monitored along the Puzi River, a watershed in which *Legionella* were frequently detected by our laboratory. The same question was imagined while analyzing the *Legionella* communities in the water samples of Puzi River, hence necessitating the use of nested PCR-DGGE with *Legionella* specific primers. The similar or same tactic has been successfully used in the detection of bacterial, bifidobacterial, sulfate-reducing bacterial and ammonia-oxidizing bacterial communities [16, 20–22]. The DGGE pattern of three step nested PCR product was found to be raised in band intensity. In spite of the fact that many artificial PCR amplicons were able to generate false signals leading confounded projection during DGGE electrophoresis, the confirmation in followed sequencing procedure can right the wrong judge and make this technique a validated method for detecting of genus *Legionella spp* [12, 23].

The selection of the primers GCLEG448 and LEG858, which produces DNA fragments apposite for DGGE pattern analysis, was completed because all DNA fragments gained with the *Legionella* specific primer pairs in the second step PCR amplification included the target positions for these DGGE primer pairs. This primer amplified a major DNA fragment with gene sequence information, provided good results as well. However, the DGGE pattern of *Legionella* is different. The lowest amount of bands was watched in the DGGE pattern of *Legionella*, indicating a low diversity inside this *Legionella* of river water sample. Because *Legionella* could not be amplified through direct amplification, no band in the total *Legionella* community DGGE pattern could be recognized as appertaining to the *Legionella* species. Due to low diversity and low number within this *Legionella* species, resulting in no or too little PCR product to give no visible bands.

The *Legionella*-specific and bacterial-*Legionella*-specific DGGE pattern gained from the Puzi River water samples displayed the existence of only two *Legionella* main species. Detection of *Legionella* connected bands from the identical samples when *Legionella* specific primer pairs were used substantiated that the DGGE band pattern does not reveal the real diversity of but only numerically major species in the river water sample. DGGE analysis of two and three step nested PCR amplification products from the same river water sample showed six major bands of which band B3 and D3 (lane B and D, Fig 2) seemed to be the most major. The other four sequences (lane B and D; band B1, B2, D1 and D2) also appeared principal members of *Legionella* community. Bands B2 and D2 had 98% sequence identity to uncultured *Legionella* sp., and bands B1, B3, D1, and D3 had 98% sequence identity to *Legionella pneumophila*. The DGGE pattern in same river water sample (Fig 2, lane C) did not display numerous major bands. Only a little weak band was visible, of which three could be sequenced. In addition, lane E did show two major bands. The gene sequencing displayed that the DGGE band patterns belonged to *Legionella pneumophila* and uncultured *Legionella* species. After summarizing the sequencing results and the DGGE patterns of corresponding samples, our results confirmed the validity of the DGGE-derived method. More than half of DGGE bands were found to have the clinically associated species of *Legionella pneumophila*. These results confirmed the infection risk of *Legionella pneumophila* in aquatic environments.

## Conclusion

Each species of *Legionella* was found to have its own bands pattern after DGGE. The three step nested PCR-DGGE method made it a very useful to study the diversity of *Legionella* with high-resolution in low concentration in river water samples. Moreover, the specificity of the PCR primer pairs targeting various phylogenetic tree of *Legionella* is of primary significance for the achievement of this method. With advantages such as cloning-independence, economic, and the high-throughput procedure, DGGE may be potential for rough specie of unknown *Legionella* or even other pathogens in a great amount of environmental samples.

## References

[1] WHO. *Legionella* and the prevention of Legionellosis. World Health Organization, Geneva; 2007.

[2] Liu YC, Cheng CD, Shi FW, Huang WK, Wang JH Legionnaires' disease—a case report. J Formosan Med Assoc. 1985; 1180–1185. 3866833

[3] Adeleke AA, Fields BS, Benson RF, Daneshvar MI, Pruckler JM, Ratcliff RM, et al *Legionella drozanskii* sp nov., *Legionella rowbothamii* sp nov and *Legionella fallonii* sp nov.: three unusual new *Legionella* species. Int J Syst Evol Micr. 2001; 51: 1150–1160. 10.1099/00207713-51-3-1151 11411684

[4] Drozanski WJ. Fatal bacterial infection in soil amoebae. Acta Microbiol Pol. 1956; 5: 315–317. 13410554

[5] Drozanski WJ. *Sacrobium lyticum* gen. nov., sp. nov., an obligate intracellular bacterial parasite of small free-living amoebae. Int J Syst Bacteriol. 1991; 41: 82–87.

[6] La Scola B, Mezi L, Weiller PJ, Raoult D Isolation of *Legionella anisa* using an amoebic coculture procedure. J Clin Microbiol. 2001; 39(1): 365–366. 10.1128/JCM.39.1.365-366.2001 11136802 PMC87733

[7] Rowbotham TJ. Current views on the relationships between amoebae, legionellae and man. Isr J Med Sci. 1986; 22: 678–689. 3793451

[8] Cirillo JD, Cirillo SL, Yan L, Bermudez LE, Falkow S, Tompkins LS Intracellular growth in *Acanthamoeba castellanii* affects monocyte entry mechanisms and enhances virulence of *Legionella pneumophila*. Infect Immun. 1999; 67: 4427–4434. 10456883 10.1128/iai.67.9.4427-4434.1999 PMC96761

[9] Fritsche TR, Sobek D, Gautom RK Enhancement of in vitro cytopathogenicity by *Acanthamoeba* spp. following acquisition of bacterial endosymbionts FEMS Microbiol Lett. 1998; 166: 231–236. 9770279 10.1111/j.1574-6968.1998.tb13895.x

[10] el Fantroussi S, Mahillon J, Naveau H, Agathos SN Introduction of anaerobic dechlorinating bacteria into soil slurry microcosms and nested-PCR monitoring. Appl Environ Microb. 1997; 63: 806–811. 10.1128/aem.63.2.806-811.1997 PMC168375 9023963

[11] Erb RW, Wagner-Do¨bler I. Detection of polychlorinated biphenyl degradation genes in polluted sediments by direct DNA extraction and polymerase chain reaction. Appl Environ Microb. 1993; 59: 4065–4073. 10.1128/aem.59.12.4065-4073.1993 PMC195868 8285706

[12] Heuer H, Smalla K. Application of denaturing gradient gel electrophoresis (DGGE) and temperature gradient gel electrophoresis (TGGE) for studying soil microbial communities In van Elsas J. D, Wellington E. M. H, and Trevors J. T (ed.), Modern soil microbiology. Marcel Dekker, Inc; 1997 p. 353–373.

[13] Levesque MJ, La Boissie`re S, Thomas JC, Beaudet R, Villemur R Rapid method for detecting *Desulfitobacterium frappieri* strain PCP-1 in soil by the polymerase chain reaction. Appl Microbiol Biotechnol. 1997; 47: 719–725. 9237393 10.1007/s002530051001

[14] Pulawska J, Sobiczewski P. Development of a semi-nested PCR based method for sensitive detection of tumorigenic *Agrobacterium* in soil. J Appl Microbiol. 2005; 98(3): 710–721. 10.1111/j.1365-2672.2004.02503.x 15715875

[15] Regeard C, Maillard J, Holliger C Development of degenerate and specific PCR primers for the detection and isolation of known and putative chloroethene reductive dehalogenase genes. J Microbiol Methods. 2004; 56(1): 107–118. 14706755 10.1016/j.mimet.2003.09.019

[16] Dar SA, Kuenen JG, Muyzer G Nested PCR-denaturing gradient gel electrophoresis approach to determine the diversity of sulfate-reducing bacteria in complex microbial communities. Appl Environ Microb. 2005; 71(5): 2325–2330. 10.1128/AEM.71.5.2325-2330.2005 PMC1087575 15870318

[17] Rossen L, Norskov P, Holmstrom K, Rasmussen OF Inhibition of PCR by components of food samples, microbial diagnostic assays and DNA-extraction solutions. Int J Food Microbiol. 1992; 17(1): 37–45. 1476866 10.1016/0168-1605(92)90017-w

[18] Shen SM, Chou MY, Hsu BM, Ji WT, Hsu TK, Tsai HF, et al Assessment of *Legionella pneumophila* in recreational spring water with quantitative PCR (Taqman) assay. Pathog Glob Health. 2015; 109(5): 236–241. 10.1179/2047773215Y.0000000023 26184706 PMC4727576

[19] Muyzer G, de Waal E C, Uitterlinden AG Profiling of complex microbial populations by denaturing gradient gel electrophoresis analysis of polymerase chain reaction-amplified genes coding for 16S rRNA. Appl Environ Microb. 1993; 59: 695–700. 10.1128/aem.59.3.695-700.1993 PMC202176 7683183

[20] Boon N, Windt WD, Verstraete W, Top EM Evaluation of nested PCR-DGGE (denaturing gradient gel electrophoresis) with group-specific 16S rRNA primers for the analysis of bacterial communities from different wastewater treatment plants. FEMS Microbiol Ecol. 2002; 39: 101–112. 10.1111/j.1574-6941.2002.tb00911.x 19709189

[21] Phillips CJ, Harris DS, Dollhopf L, Gross KL, Prosser JI, Paul EA Effects of agronomic treatments on structure and function of ammonia-oxidizing communities. Appl Environ Microb. 2000; 66: 5410–5418. 10.1128/aem.66.12.5410-5418.2000 PMC92476 11097922

[22] Temmerman R, Masco L, Vanhoutte T, Huys G, Swings J Development and Validation of a Nested-PCR-Denaturing Gradient Gel Electrophoresis Method for Taxonomic Characterization of Bifidobacterial Communities. Appl Environ Microb. 2003; 69(11): 6380–6385. 10.1128/AEM.69.11.6380-6385.2003 PMC262296 14602589

[23] Suzuki MT, Giovannoni SJ. Bias caused by template annealing in the amplification mixtures of 16S rRNA genes by PCR. Appl Environ Microb. 1996; 62: 625–630. 10.1128/aem.62.2.625-630.1996 PMC167828 8593063

